# Silymarin Constituents Enhance ABCA1 Expression in THP-1 Macrophages

**DOI:** 10.3390/molecules21010055

**Published:** 2015-12-31

**Authors:** Limei Wang, Susanne Rotter, Angela Ladurner, Elke H. Heiss, Nicholas H. Oberlies, Verena M. Dirsch, Atanas G. Atanasov

**Affiliations:** 1 Department of Pharmacognosy, University of Vienna, Althanstrasse 14, A-1090 Vienna, Austria; limei.wang@univie.ac.at (L.W.); a0800134@unet.univie.ac.at (S.R.); angela.ladurner@univie.ac.at (A.L.); elke.heiss@univie.ac.at (E.H.H.); verena.dirsch@univie.ac.at (V.M.D.); 2 Department of Chemistry & Biochemistry, University of North Carolina at Greensboro, Greensboro, NC 27402, USA; n_oberli@uncg.edu

**Keywords:** ABCA1, silymarin, isosilybin A, cholesterol efflux, macrophages, flavonolignans

## Abstract

Silymarin is a hepatoprotective mixture of flavonolignans and flavonoids extracted from the seeds of milk thistle (*Silybum marianum* L. Gaertn). This study investigates the effect of major bioactive constituents from silymarin, silybin A, silybin B, isosilybin A, isosilybin B, silydianin, silychristin, isosilychristin, and taxifolin, on the expression of ABCA1, an important cholesterol efflux transporter, in THP-1-derived macrophages. Four of the studied compounds, isosilybin A, silybin B, silychristin and isosilychristin, were found to significantly induce ABCA1 protein expression without affecting cell viability. Moreover, isosilybin A, a partial PPARγ agonist, was found to promote cholesterol efflux from THP-1 macrophages in a concentration-dependent manner. These findings first show ABCA1 protein up-regulating activity of active constituents of silymarin and provide new avenues for their further study in the context of cardiovascular disease.

## 1. Introduction

Natural products have proven their potential as a source for development of very successful therapeutics [1]. Silymarin is the main bioactive component extracted from the seeds of milk thistle, *Silybum marianum* (L.) Gaertn. (Asteraceae), which is used as a hepatoprotective agent. Silymarin is a standardized mixture of flavonolignans, and mainly composed of silybin (synonymous with silibinin), isosilybin, silydianin and silychristin, and small amounts of other phenolic compounds, such as quercetin, dehydrosilybin, isosilychristin and taxifolin [2,3,4]. Silybin is a mixture of silybin A and silybin B, while isosilybin is a mixture of isosilybin A and isosilybin B [5]. The main use of silymarin is focused on its hepatoprotective properties. A meta-analysis of five trials with in total 602 patients with (alcoholic) liver cirrhosis found that silymarin significantly reduced liver-related mortality by around 7% compared with placebo treatment [6]. Besides, silymarin was proposed as a potential hypocholesterolaemic agent protecting fatty liver development caused by disordered lipid metabolism [7]. Furthermore, silymarin significantly improved plasma lipoprotein profile, e.g., by increasing the levels of high density lipoprotein (HDL) and decreasing the levels of VLDL and triacylglycerol (TAG) in rats fed cholesterol-rich diet. Moreover, silymarin positively influences liver lipid content, inhibits cholesterol absorption and thereby counteracts the development of fatty liver, rationalizing its use to combat hypercholesterolemia overall [8,9,10]. Although silymarin is generally prescribed as a hepatoprotective agent in a range of different conditions, it should be noted that its efficacy in some liver-related disorders is still debated. A recent randomized controlled trial failed to demonstrate effectiveness of silymarin supplementation on liver disease in patients with chronic hepatitis C unsuccessfully treated with interferon therapy [11].

The transmembrane ATP-binding cassette transporter A1 (ABCA1) protein plays a crucial role in reverse cholesterol transport (RCT), by transferring intracellular cholesterol and phospholipids to lipid-poor apolipoproteins. The efflux of cholesterol from macrophages or tissues by ABCA1 is considered beneficial in the prevention of cardiovascular disease [12]. Loss of ABCA1 protein occurs in serious inherited diseases, such as Tangier disease and familial HDL deficiency, which are accompanied by decreased cholesterol efflux and atherosclerosis development [13]. A clinical study found that ABCA1 mutations are associated with increased intima-media thickness resulting from decreased cholesterol efflux [14]. Cholesterol efflux is the first step of RCT, and it comprises transmembrane transporter-mediated transfer of intracellular cholesterol to extracellular lipid-poor lipoproteins. There are three kinds of transmembrane transporter proteins with important cholesterol efflux-mediating functions, particularly ABCA1, ABCG1, and scavenger receptor class B member 1 (SR-B1). Among them, the ABCA1 transporter protein is the most important one for cholesterol transportation [15]. ABCA1 expression and subsequent cholesterol efflux are known to be enhanced by activation of the nuclear receptor peroxisome proliferator-activated receptor gamma (PPARγ) [16]. Moreover, our previous study suggested that among the active components of silymarin, isosilybin A could partially activate PPARγ [17]. Since silymarin exhibits beneficial effect on plasma cholesterol and lipoprotein levels, we hypothesized that silymarin constituents might exert a direct effect on ABCA1 expression levels. Based on this hypothesis, in this study we first investigated the ability of isosilybin A to activate cholesterol efflux and ABCA1 expression. Furthermore, we surveyed the ABCA1-inducing activity of further 8 key silymarin constituents (silybin A, silybin B, isosilybin B, silybin, silydianin, silychristin, isosilychristin and taxifolin) which are unable to activate PPARγ [18].

## 2. Results and Discussion

### 2.1. Impact of the Studied Components of Silymarin on THP-1 Cell Viability

**Figure 1:**
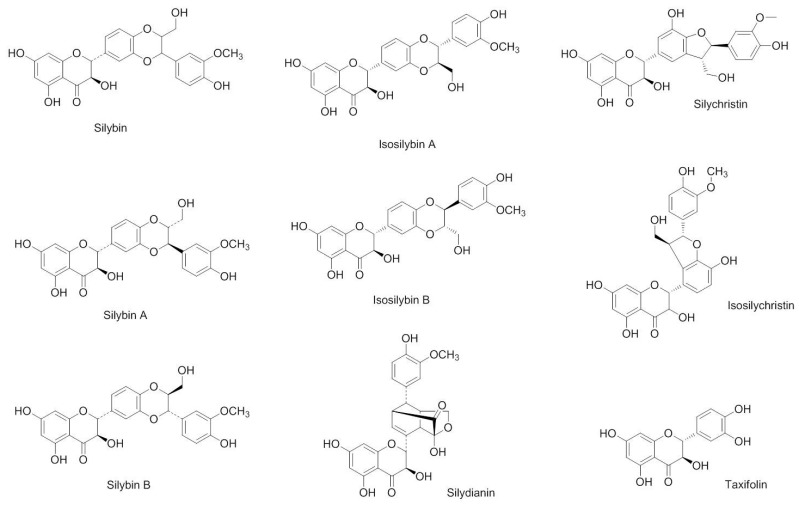
Silymarin constituents investigated in this study.

**Figure 2:**
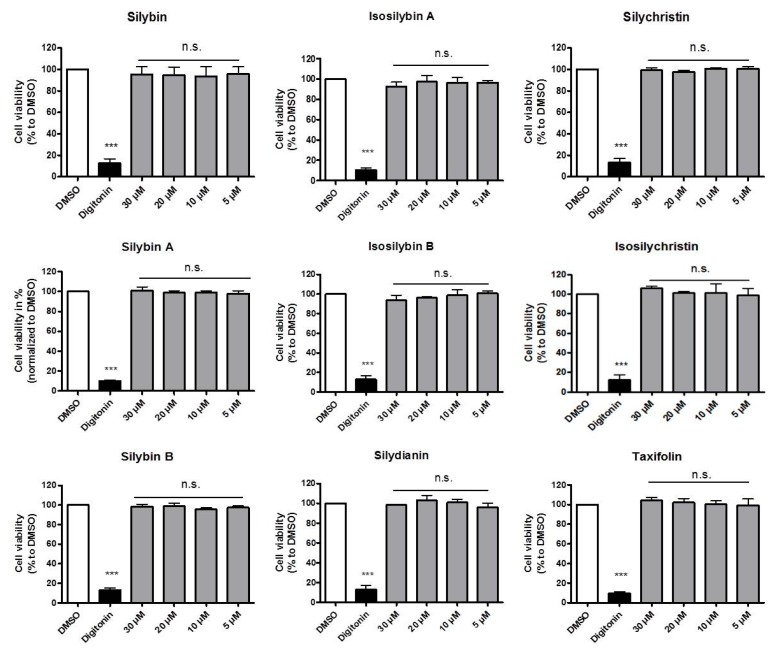
Effect of the investigated compounds on THP-1 cell viability. Resazurin conversion assay was used for the analysis of THP-1 viability. THP-1 cells were first differentiated into macrophages with 200 nM PMA for 72 h. Then, cells were treated with solvent vehicle control (DMSO), 50 μg/mL digitonin and the indicated compounds from 5 to 30 μM for 24 h. After the treatment, THP-1 macrophages were washed and incubated with 10 μg/mL resazurin for another 4 h. Fluorescence of converted resorufin was measured and the increased fluorescent signal was quantified as cell viability. Experiments were performed three times in triplicate. All values are mean ± SD (*n* = 3) compared to the solvent vehicle control (DMSO), n.s.: not significant, *** *p* < 0.001 (ANOVA/Bonferroni).

The resazurin conversion assay is a simple and versatile method of measuring mammalian cell viability that correlates with the conversion of the nonfluorescent resazurin to highly fluorescent resorufin [19,20]. To exclude that the nine studied silymarin constituents (silybin A, silybin B, isosilybin A, isosilybin B, silybin, silydianin, silychristin, isosilychristin and taxifolin) (Figure 1) affect the viability of the used THP-1 cells, we have first measured their ability to affect resazurin conversion. Digitonin, a natural cytotoxic product, was used as positive control in this experiment. As presented in Figure 2, none of the compounds exhibited any effect on the viability of THP-1 macrophages in the tested concentration range (from 5 to 30 μM), whereby digitonin, tested at 40.7 μM (equaling 50 μg/mL), decreased THP-1 cell viability by around 90%.

### 2.2. Impact of Isosilybin A on Cholesterol Efflux

**Figure 3:**
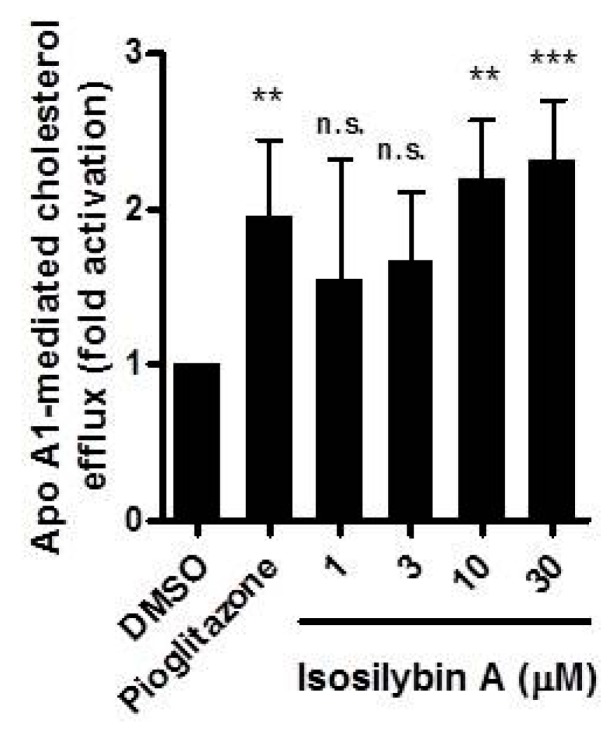
Effect of isosilybin A, a partial PPARγ agonist, on apo A1-mediated cholesterol efflux. First, THP-1 monocytes were differentiated into adherent macrophages by treatment with 200 nM PMA for 72 h. The differentiated THP-1 macrophages were loaded with [^3^H]-cholesterol together with solvent vehicle control (DMSO), pioglitazone (10 μM) and varying concentrations of isosilybin A (from 1 to 30 μM) in serum-free medium with 0.1% fatty acid free BSA and 10 μg/mL unesterified cholesterol for 24 h to achieve equilibration of the intracellular cholesterol pool. After that, cells were incubated with the same treatments with or without 10 μg/mL apo A1 dissolved in serum-free medium for 6 h. The [^3^H]-cholesterol released into the medium as well remaining in the cell lysates was quantified by liquid scintillation counting. Three independent experiments were performed. All values are mean ± SD (*n* = 3) compared to the solvent vehicle control (DMSO), n.s.: not significant, ** *p* < 0.01, *** *p* < 0.001 (ANOVA/Bonferroni).

Knowing isosilybin A as a partial PPARγ agonist [17] and PPARγ as activator of macrophage cholesterol efflux, we were prompted to survey cholesterol efflux in human THP-1-derived macrophages after treatment with isosilybin A. As shown in Figure 3, isosilybin A concentration-dependently induced cholesterol efflux from 1 to 30 μM (EC_50_ = 4.1 μM), mimicking the effect of pioglitazone (10 μM), a well-established PPARγ agonist, that was used as a positive control [21].

### 2.3. Impact of the Active Components of Silymarin on ABCA1 Protein Expression

**Figure 4:**
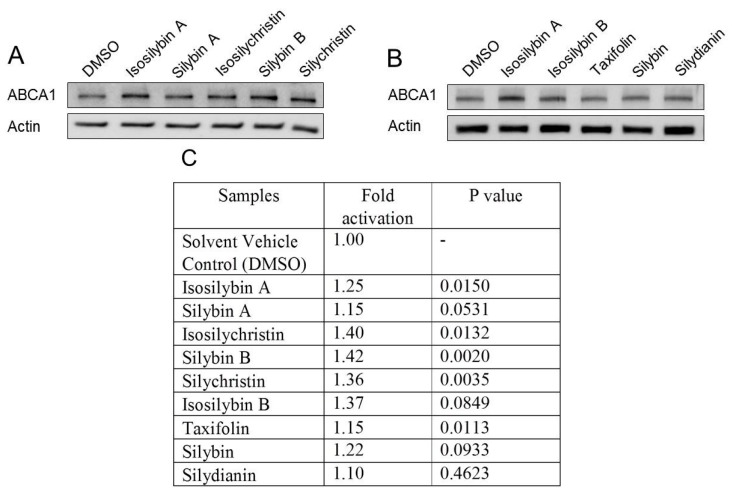
Effect of the investigated compounds on ABCA1 protein levels. Western blot analysis shows ABCA1 protein expression levels. THP-1 macrophages were treated with solvent control (DMSO) or the investigated compounds (10 μM). After 24 h incubation, cells were lysed and proteins analyzed via SDS-PAGE. (**A**) presents the compounds silybin A, isosilychristin, silybin B and silychristin; (**B**) presents the compounds isosilybin B, taxifolin, silybin and silydianin. Experiments are independently performed three times, and representative images are shown; The summarized results from the quantification of all performed experiments are presented in (**C**). All values are mean (*n* = 3) compared to the solvent vehicle control (DMSO), two-tailed *t*-test was used for statistics.

PPARγ agonists increase cholesterol efflux through up-regulation of ABCA1 protein levels [16,21]. Therefore, we next investigated whether isosilybin A leads to ABCA1 up-regulation. Next to isosilybin A, we also evaluated eight other silymarin constituents (at 10 μM), which are not PPARγ agonist [18]. Figure 4 presents the results obtained upon quantification of the ABCA1 protein expression levels. As expected, isosilybin A (10 μM) induced a significant up-regulation of the ABCA1 protein expression, showing a 1.25-fold activation compared to the solvent vehicle control (DMSO, 0.1%) treated cells. Interestingly, besides isosilybin A, four of the other investigated compounds, silybin B, silychristin, isosilychristin, and taxifolin, also induced ABCA1 expression.

Notably, some of the tested structurally similar compounds exhibit a quite distinct ABCA1-inducing potential. For example, silybin B significantly induced ABCA1 protein expression with a 1.42-fold activation, whereas silybin A exerted no effect. As a mixture of silybin A and silybin B, silybin showed slight up-regulation of ABCA1 protein level with a 1.22-fold activation. Therefore, the difference in stereochemistry appears crucial for the ABCA1-inducing activity. However, unlike the pair of silybin A and silybin B, silychristin and isosilychristin showed almost the same effect, upregulating ABCA1 1.36- and 1.40-fold, respectively. In this case, the stereochemical difference between silychristin and isosilychristin seems not crucial for the ABCA1 protein up-regulation in THP-1 macrophages. Taxifolin (dihydroquercetin) is a flavonoid with simpler chemical structure compared with the other active components of silymarin. Though its effect is not strong (1.15-fold activation), it significantly increased the ABCA1 protein expression in THP-1 macrophages. In our previous study, taxifolin even decreased PPARγ activity [17], indicating overall the existence of alternative (different than PPARγ activation) ABCA1 expression-enhancing mechanisms affected by the silymarin constituents in our cell model.

PPARγ activation is a very well established enhancer of ABCA1 expression leading to increased macrophage cholesterol efflux. The enhanced efflux might therefore be due to PPARγ-activation upon isosilybin A treatment, although the existence of additional molecular mechanisms of action cannot be ruled out. Moreover, a causal link between increased ABCA1 level and increased efflux is not directly demonstrated in our work. Furthermore, since silybin B, silychristin, isosilychristin, and taxifolin are not active or even inhibit PPARγ, their ABCA1-enhancing effects are likely mediated through mechanisms other than PPARγ activation.

## 3. Materials and Methods

### 3.1. Chemicals, Cell Culture Reagents and Antibodies

Pioglitazone was bought from Molekula (Munich, Germany) and [^3^H]-cholesterol (1 mCi, 37 MBq) from Perkin Elmer Life Sciences (Vienna, Austria). Digitonin was purchased from Sigma-Aldrich via Fluka (Vienna, Austria). Bovine serum albumin (BSA) was obtained from Roth (Karlsruhe, Germany). Resazurin sodium salt, water soluble unesterified cholesterol, apolipoprotein (apo) A1, and phorbol 12-myristate 13-acetate (PMA) were provided by Sigma-Aldrich.

Phenol red Roswell Park Memorial Institute (RPMI) 1640 medium without l-glutamine was purchased from Lonza (Basel, Switzerland). Fetal bovine serum (FBS) was from Gibco (Lofer, Austria).

Primary antibody against ABCA1 was provided by Novus Biologicals (Vienna, Austria). The anti-actin antibody was obtained from MP biologicals (Illkirch, France). HRP-linked anti-rabbit IgG secondary antibody was acquired from New England Biolabs (Frankfurt, Germany) and horseradish peroxidase conjugated goat anti-mouse secondary antibody from Upstate (Millipore, Vienna, Austria). All antibodies were diluted 1:500 except the anti-mouse secondary antibody (1:10,000).

### 3.2. Sources of the Compounds

The sources of the compounds were described in detail previously [17]. Isosilybin A and its derivatives were dissolved in dimethyl sulfoxide (DMSO) at 30 mM, aliquoted and stored at −20 °C until use. An equal amount of DMSO was used for each condition in all experiments and the final DMSO concentrations did not exceed 0.1% to assure that the solvent vehicle does not influence the results itself.

### 3.3. Cell Culture

The human THP-1 monocytic cells were obtained from ATCC^®^ and grown in RPMI-1640 medium supplemented with 2 mM glutamine, 100 U/mL benzylpenicillin, 100 μg/mL streptomycin and 10% heat-inactivated FBS,. The THP-1 cells were cultured in T175 flasks at 5% CO_2_ and 37 °C, and split every two days to assure that the concentration is not exceeding 0.8 × 10^6^ per mL (to assure cell viability > 95%).

### 3.4. Resazurin Conversion Assay

The assay was performed as previously described with slight modifications [20]. THP-1 cells were seeded in 96-well plates at a density of 0.2 × 10^6^ per mL in a volume of 100 μL per well. THP-1 macrophages were acquired by treatment with 200 nM PMA for 72 h in RPMI-1640 medium supplemented with 10% heat-inactivated FBS. After differentiation, THP-1 macrophages were washed once with warm PBS (37 °C) and treated with the indicated compounds at 5, 10, 20 and 30 μM in RPMI-1640 FBS-free medium supplemented with 0.1% BSA and 10 μg/mL unesterified cholesterol. The saponine digitonin, was used as positive control (50 μg/mL). After 24 h incubation with the compounds, cells were washed once with warmed PBS again and incubated with 10 μg/mL resazurin which is dissolved in PBS to allow the conversion to the highly fluorescent resorufin. After 4 h incubation, the plates were read with a Tecan GENiosPro plate reader (Männedorf, Switzerland) at a wavelength of 580 nm (emission) and 535 nm (excitation).

### 3.5. ABCA1 Protein Quantification

Western blot analysis was used for relative ABCA1 protein quantification. Briefly, cells were seeded in 6-well plates at a density of 0.2 × 10^6^ per mL with a volume of 4 mL for each well. THP-1 monocytes were differentiated into macrophages in RPMI-1640 medium supplemented with 10% heat-inactivated FBS in the presence of 200 nM PMA for 72 h. After differentiation, cells were washed once with pre-warmed PBS and loaded with the indicated compounds at 10 μM. Cells were incubated with the respective compounds for 24 h, a period of time that appeared in pilot experiments as optimal for ABCA1 induction by PPARγ agonists such as pioglitazone. Cells were then washed once with cold PBS and then lysed with NP40 buffer (150 mM NaCl; 50 mM HEPES (pH 7.4); 1% NP40; 1% protease inhibitor Complete™ (Roche); 1% phenylmethylsulfonyl fluoride (PMSF); 0.5% Na_3_VO_4_; 0.5% NaF) for 30 min at 4 °C. The lysed cells were collected with a scratcher and centrifuged for 20 min at 16,060 g. Cell pellets were discarded and the supernatant was transferred into a new Eppendorf tube for total protein quantification using a Bradford assay. Twenty micrograms of protein was resolved via 10% SDS polyacrylamide gel electrophoresis (SDS-PAGE) for 80 min at 25 mA per gel for separation. The fractionated protein was analyzed with antibodies against ABCA1 and actin, respectively. Protein bands were visualized with ECL reagent and a LAS-3000 luminescent image analyzer (Fujifilm, Düsseldorf, Germany) using AIDA image analyzer 4.06 software (Raytest, Sprockhövel, Germany).

### 3.6. Cholesterol Efflux Assay

Human THP-1 monocytic cells were seeded in 24-well plates at a density of 0.2 × 10^6^ per mL with a volume of 1 mL for each well. Cells were differentiated into adherent macrophages with 200 nM PMA for 72 h in RPMI-1640 medium containing 10% FBS. THP-1 cells were washed once with pre-warmed PBS and loaded with 0.3 μCi/mL [^3^H]-cholesterol together with 10 μM pioglitazone or varying concentrations of isosilybin A in serum-free RPMI-1640 medium containing 0.1% BSA and 10 μg/mL unesterified cholesterol. After equilibrating the cholesterol pools for 24 h, THP-1 macrophages were washed twice with warmed PBS and incubated in serum-free RPMI-1640 medium with the same compounds (pioglitazone or isosilybin A) in the presence and absence of apo A1 (10 μg/mL) for 6 h. The [^3^H]-cholesterol released into the medium (extracellular radioactivity) and maintained in the cell lysates (intracellular radioactivity) were quantified by liquid scintillation counting. The assays were performed in triplicate and the percent apo A1-mediated cholesterol efflux was calculated as follows:

Apo A1 mediated cholesterol efflux = [((extracellular cpm) apo A1/(intracellular cpm + extracellular cpm) apo A1) × 100] − [((extracellular cpm) no apo A1/(intracellular cpm + extracellular cpm) no apo A1) × 100].

### 3.7. Statistical Analysis

All experiments in this study were performed at least three times independently. Figures with bar graphs are presented as mean ± SD. One-way analysis of variance (ANOVA) and *t*-tests were used for statistical analysis using GraphPad Prism software version 4.03 (GraphPad Software Inc., La Jolla, CA, USA). The differences were considered significant when *p* < 0.05, indicated with one asterisk (*). Further details for each experiment can be found in the respective figure legends.

## 4. Conclusions

In summary, we identify the silymarin constituents isosilybin A, silybin B, silychristin, isosilychristin, and taxifolin to increase ABCA1 expression in THP-1-derived macrophages. In addition, isosilybin A promotes cholesterol efflux from macrophages in a concentration-dependent manner, possibly due to its PPARγ-activating properties. Overall, our study provides for the first time evidence for ABCA1-upregulating effects of silymarin constituents, providing a possible link to its described hypocholesterolaemic *in vivo* action, and opening new research directions that might help to better understand molecular mechanisms underlying the action of this important health-promoting herbal product.
